# PET/CT-based radiomics analysis may help to predict neoadjuvant chemotherapy outcomes in breast cancer

**DOI:** 10.3389/fonc.2022.849626

**Published:** 2022-11-07

**Authors:** Liping Yang, Jianfei Chang, Xitao He, Mengye Peng, Ying Zhang, Tingting Wu, Panpan Xu, Wenjie Chu, Chao Gao, Shaodong Cao, Shi Kang

**Affiliations:** ^1^ Department of Positron Emission Tomography-Compute Tomography (PET-CT), Harbin Medical University Cancer Hospital, Harbin, China; ^2^ Department of Chinese Medicine, Qingdao West Coast New Area People's Hospital, Qingdao, China; ^3^ Anesthesiology Department, Second Hospital of Harbin City, Harbin, China; ^4^ Medical Imaging Department, The Fourth Affiliated Hospital of Harbin Medical University, Harbin, China; ^5^ Medical Imaging Department, The Second Hospital of Heilongjiang Province, Harbin, China

**Keywords:** breast neoplasms, Positron Emission Tomography-Computed Tomography, neoadjuvant therapies, pathological complete response, artificial intelligence

## Abstract

**Background:**

The aim of this study was to evaluate the clinical usefulness of radiomics signature-derived ^18^F-fluorodeoxyglucose (^18^F-FDG) positron emission tomography–computed tomography (PET-CT) for the early prediction of neoadjuvant chemotherapy (NAC) outcomes in patients with (BC).

**Methods:**

A total of 124 patients with BC who underwent pretreatment PET-CT scanning and received NAC between December 2016 and August 2019 were studied. The dataset was randomly assigned in a 7:3 ratio to either the training or validation cohort. Primary tumor segmentation was performed, and radiomics signatures were extracted from each PET-derived volume of interest (VOI) and CT-derived VOI. Radiomics signatures associated with pathological treatment response were selected from within a training cohort (*n* = 85), which were then applied to generate different classifiers to predict the probability of pathological complete response (pCR). Different models were then independently tested in the validation cohort (*n* = 39) regarding their accuracy, sensitivity, specificity, and area under the curve (AUC).

**Results:**

Thirty-five patients (28.2%) had pCR to NAC. Twelve features consisting of five PET-derived signatures, four CT-derived signatures, and three clinicopathological variables were candidates for the model’s development. The random forest (RF), k-nearest neighbors (KNN), and decision tree (DT) classifiers were established, which could be utilized to predict pCR to NAC with AUC ranging from 0.819 to 0.849 in the validation cohort.

**Conclusions:**

The PET/CT-based radiomics analysis might provide efficient predictors of pCR in patients with BC, which could potentially be applied in clinical practice for individualized treatment strategy formulation.

## Introduction

The pathological staging system suggested by the International Association is taken as the reference standard in therapeutic strategy decision for patients with breast cancer (BC). Stages IIB and IIIC BC are categorized as locally advanced stages, and radical surgical resection is not currently preferred (1). Presently, neoadjuvant chemotherapy (NAC) is recommended for patients with these cancers. Thus, NAC has become a standard option for potentially surgically resectable BC (2). As a preoperative treatment plan, the main advantage of NAC lies in reducing the size of the primary tumor and down-staging the tumor burden before surgery (3).

To date, pCR has been used as an alternative prognostic endpoint in clinical trials of neoadjuvant drugs for BC patients (4). A series of works have investigated the potential association of pCR with the long-term survival outcomes in patients with BC. An earlier randomized clinical trial revealed that pCR was correlated with prolonged disease-free survival (DFS) in BC (5). Another previous study has demonstrated that pCR in human epidermal growth factor receptor 2 (Her-2)-positive BC is associated with substantially longer times to recurrence and death (6). However, other clinical trials indicated that there was no significant benefit in terms of overall survival (OS) (*p* = 0.51) and recurrence-free survival (*p* = 0.80) between the pCR and non-pCR groups (7). The conflicting results achieved from different studies might raise strong demands for a biomarker that could be applied to select candidates who would derive added benefit from NAC treatment.

Currently, the therapeutic effect during NAC (pCR or non-pCR) was mainly evaluated through pathological analysis of surgical specimens at the end of NAC, but it failed to reflect the tumor changes in the early stage and to monitor the treatment response in real time (8). In contrast, imaging examinations are noninvasive and reproducible. A pCR with NAC can be assessed with various imaging modalities, such as mammography, breast ultrasound, magnetic resonance imaging (MRI), and positron emission tomography-computed tomography (PET-CT). Several clinical trials and meta-analyses have investigated the diagnostic efficacy of various imaging modalities after NAC treatment and compared the accuracy of preoperative measurements with the final pathologic size of the tumor; however, there is no conclusion yet regarding the most reliable and accurate modality (9–11). Many of them have shown MRI to be highly sensitive but rather have low specificity for identifying pathological complete response (pCR = ypT0N0). Another previous study has shown that PET-CT may more accurately predict the pCR because of the functional imaging ability for viable tumor cells compared with anatomic tumor size (12). However, there remains a shortage of reliable clinical pCR indicators based on conventional imaging modalities due to the great heterogeneity of BC. Radiomics analysis provides significant clinical usefulness and enables researchers to non-invasively assess tumor heterogeneity, which is an important step towards personalized treatment (13, 14). To that end, radiomics shows great prospects in evaluating treatment response of NAC regimens in patients with BC. However, the current status is that almost all previous works concentrated on x-ray, computed tomography, and MRI. Very few radiomics studies have involved the predictive value of PET-CT imaging (15, 16). The goal of this study was to develop and validate radiomics predictive models for personalized pCR assessment during NAC in patients with BC.

## Materials and methods

### Study population

Specific inclusion criteria were listed as follows: (i) histological diagnosis of primary BC, (ii) performance of ^18^ F-FDG PET/CT for staging purposes before any treatment, (iii) NAC as primary treatment followed by surgery, and (iv) a single lesion with a maximum diameter ≥ 1 cm and had no difficulty in tumor margin delineation. The research protocol was reviewed, approved, and overseen by the institutional review board of Harbin Medical University Cancer Hospital. Informed consent permission was not required in line with the local ethics committee’s regulations for retrospective research.

### Image acquisition

PET/CT images were acquired using the Discovery VCT 64 PET/CT system (GE Healthcare, Milwaukee, USA). All patients were requested to fast for 4–6 h prior to PET-CT scans. In addition, there are strict regulations on the blood glucose level of each patient, which must be controlled below 11.1 mmol/L ahead of ^18^F-FDG, which is injected intravenously. All patients must lie still and rest for at least 1 h before starting the scans after an injection of 7.4 MBq (0.2 mCi)/kg ^18^F-FDG. Firstly, low-dose CT scans (free-breathing state and unenhanced images) were performed before whole-body PET-CT examination. Image reconstruction was performed based on the 3D ordered subset expectation–maximization algorithm (two iterations and 17 subsets). The baseline PET-CT scans were performed before NAC administration, and all PET-CT examinations were completed in the case of the same institution with the same equipment and acquisition parameters, which were listed as follows: tube voltage, 140 kV; tube current, 150 mA; slice thickness, 3.75 mm; matrix size, 512 × 512; and field of view, 450 mm.

### Image analysis

Image analyses were performed using an advanced post-processing software (PET VCAR; GE Healthcare). Two nuclear medicine physicians with more than 10 years of diagnostic experience, blinded to the outcome of surgery and pathology, independently assessed the images. Final results were re-checked by a senior radiologist and any disagreement was settled by discussion. Each PET-derived volume of interest (VOI) was defined with a threshold of 40% of the maximum standardized uptake value (SUVmax), and then corresponding metabolic parameters were automatically calculated by PET VCAR software.

### NAC regimen and pathological assessment

A paclitaxel-based NAC regimen was performed in 112 patients (90.3%). As for the remaining 12 patients (9.7%), a recommended NAC protocol with anthracycline plus paclitaxel was administrated. Anti-Her2 therapeutic strategy (trastuzumab, starting dose of 8 mg/kg, maintenance dose of 6 mg/kg) was added for patients with Her2 amplification. Surgery was performed within 4 weeks of the end of NAC. According to the routine pathological results when NAC treatment was completed, corresponding pathological response to NAC was assessed by one pathologist with more than 10 years of work experience. No residual invasive cancer was identified in the initial lesion area and both axillary lymph nodes after surgery resection, which was defined as pCR; otherwise, non-pCR (17).

### Image segmentation and feature extraction

**Figure 1:**
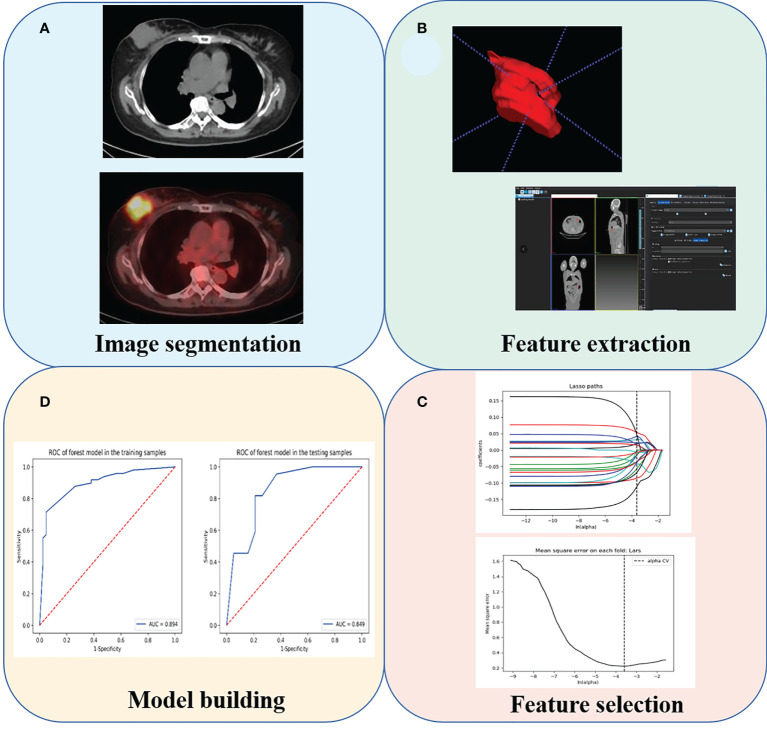
The radiomics analysis workflow. **(A)** Tumor segmentation. **(B)** Feature extraction. **(C)** Feature selection. **(D)** Model building.

An overview of radiomics workflow is displayed in **Figure 1**. The tumor lesion was delineated on axial PET and CT images using LIFEx software (open-source software; www.lifexsoft.org/index.php). A VOI that covered the entire tumor was delineated by segmentation on each axial slice of CT and PET. All 3D segmentation was first delineated automatically by means of thresholding or clustering, which were corrected by a radiologist manually afterwards. The VOI of the breast lesion was defined on PET images with a threshold of 40% of the SUVmax. Tumor segmentation was done by a nuclear medicine physician with more than 15 years of diagnostic experience in BC, blinded to surgical and pathological results.

We adopted three steps to preprocess the PET and CT images prior to feature extraction. Firstly, we resampled all images to a uniform voxel size of 1 mm × 1 mm × 1 mm using linear interpolation to minimize the influence of different layer thicknesses. Secondly, based on the gray-scale discretization process (bin width for CT = 25, bin width for PET = 0.1), we convert the continuous image into discrete integer values. Finally, we use the Laplacian of Gaussian and wavelet image filters to eliminate the mixed noise in the image digitization process in order to obtain low- or high-frequency features. Radiomics signatures were extracted from each PET-derived VOI and CT-derived VOI by applying dedicated AK software (Artificial Intelligence Kit; GE Healthcare, China, Shanghai). Each radiomic signature was applied with a *Z*-score normalization to transform the data into standardized intensity range. All patients enrolled were randomly assigned in a 7:3 ratio to either the training cohort or validation cohort. Synthetic minority oversampling technique was adopted due to the imbalance number of pCR- and non-pCR patients in the training cohort. Next, the feature selection was carried out within the training cohort by using a step-by-step selection method.

### Radiomics signature selection

After the radiomics features extraction, all missing data were replaced by the median value in the training set. *Z*-score normalization was done on each radiomics feature. In addition, the same preprocessing procedure was also applied to the validation set. Intra- and inter-class correlation coefficients (ICCs) were computed to evaluate the intra- and inter-observer reproducibility of radiomics signature extraction. For the 40 cases of PET-derived and CT-derived VOIs selected randomly (20 cases of pCR to NAC and 20 cases of non-pCR to NAC), radiologists A and B extracted the signatures independently. All radiomics signatures were re-extracted by radiologist A 2 weeks later, and radiomics signatures with ICC lower than 0.80 were considered as the poor reproducibility of the signature and then were excluded.

After the intra- and inter-operator agreement evaluation, radiomic features with ICC > 0.80 were selected for further analysis. Next, the following three steps were carried out within the training cohort to screen radiomic features related to pathological status after receiving NAC therapy. Firstly, univariate logistic regression analysis test was applied to select features with *p*-value < 0.05 for the subsequent analysis. Secondly, multivariate logistic regression analysis was utilized to choose features closely related to pathological status. Finally, a subset of the most robust and non-redundant radiomic signatures was retained using the least absolute shrinkage and selection operator (LASSO) method.

### Models building and predictive performance assessment

All cases in the training set were used to train the predictive model, while cases in the test set were utilized to independently evaluate the model’s performance. Three different machine learning classifiers, namely, k-nearest neighbors (KNN), random forest (RF), and decision tree (DT), were developed separately. All radiomics models were trained in the training cohort, and then tested in the validation cohort. The predictive performance of the developed models was assessed using receiver operating characteristic (ROC) curve.

### Statistical analysis

Statistical analysis was performed using R-studio and GraphPad Prism software. Radiomics parameters between pCR group and non-pCR groups were tested by Mann–Whitney *U* test. Statistical analysis was performed using SPSS software (version 23.0, Chicago, IL, USA). In addition, two-sided *p*-value below 0.05 was considered statistically significant.

## Results

### Patient demographics and pathological outcomes

A total of 124 patients who met the inclusion and exclusion criteria shown above were studied. The baseline demographic characteristics are displayed in **Table 1**. There were 85 cases (24 patients with pCR and 61 with non-pCR) in the training group and 39 cases (11 patients with pCR and 28 with non-pCR) in the validation group. In univariate logistic regression analysis and multivariate logistic regression analysis, three parameters, namely, Ki-67, tumor grade, and TLG, were demonstrated to be independent predictors of pCR by multivariate logistic regression analysis (**Supplementary Table 1**).

**Table 1:** Demographic information and clinicopathological characteristics of selected patients with NSCLC.

Variable	Training cohort (*n* = 85)	Validation cohort (*n* = 39) *p*-value
**Sex, *n* (%)**		0.334
Female	83 (97.65)	39 (100%)
Male	2 (2.35%)	0 (00.00%)
**Age (years)**	28.00 (23.75, 39.00)	31 (25.75, 46.00)0.452
**Tumor Histology, *n* (%)**		0.199
Invasive ductal carcinoma	64 (75.29%)	25 (64.10%)
Invasive lobular carcinoma	21 (24.71%)	14 (35.90%)
**Tumor Grade, *n* (%)**		0.986
Moderately differentiated	32 (37.65%)	15 (38.46%)
Poorly differentiated	30 (35.29%)	14 (35.90%)
Well differentiated	23 (27.05%)	10 (25.64%)
**Pathological T stage, *n* (%)**		0.859
1	0 (00.00%)	0 (00.00%)
2	18 (21.18%)	10 (25.64%)
3	23 (27.06%)	10 (25.64%)
4	44 (51.76%)	19 (48.72%)
**Pathological N stage, *n* (%)**		0.030
2	42 (49.41%)	12 (30.77%)
3	23 (27.06%)	20 (51.28%)
4	0 (00.00%)	0 (00.00%)
**Molecular subtype, *n* (%)**		0.379
Luminal A	7 (8.24%)	3 (7.69%)
Luminal B	10 (11.76%)	9 (23.08%)
HER2	38 (44.71%)	13 (33.33%)
TNBC	30 (35.39%)	14 (35.90%)
**ER (%)** **Her-2 status, *n* (%)**	76.00 (0.00, 90.00)	72.00 (5.00, 80.00) 0.348
Positive	40 (47.06%)	20 (51.28%) 0.662
Negative	45 (52.94%)	19 (48.72%)
**PR (%)**	14.00 (0.00, 75.00)	16.00 (5.00, 80.00) 0.552
**Ki-67 (%)**	20.00 (10.00, 50.00)	15.00 (5.00, 40.00) 0.249
**CEA (ng/ml)**	2.73(1.34, 5.20)	2.55 (1.34, 4.95) 0.496
**CAI53 (ng/ml)**	10.95 (7.42, 14.25)	12.10 (8.22, 16.65) 0.310
**SUVmax**	5.65 (2.96, 7.85)	5.40 (2.60, 7.79) 0.420
**SUVmean**	3.87 (3.36, 5.42)	3.79 (3.19, 5.04) 0.395
**TLG**	20.95 (9.03, 52.68)	22.01 (9.03, 58.75) 0.406

Continuous variables are expressed as median (IQR). SUVmax, maximum standardized uptake value; SUV mean, mean standardized uptake value; TLG, total lesion glycolysis; CEA, carcinoembryonic antigen; CA153, carbohydrate antigen 153; ER, estrogen receptor; PR, progesterone; TNBC, triple-negative breast cancer; Her-2, human epidermal growth factor receptor 2; Ki-67, antigen Ki-67.

The median follow-up duration was 14.7 months (range, 4.2–25.9 months). When the follow-up ended, one patient died and seven patients had disease progression. pCR to NAC treatment was observed in 35 patients, and the overall pathologic response rate was 28.2%. **Table 2** displays the therapeutic effect to NAC. Representative PET/CT images of a patient with pCR and a patient with non-pCR after NAC are demonstrated in **Supplementary Figure 1**.

**Table 2:** The results of pathological response of all patients.

Histopathologic response	No. of patients (%)
Pathological complete response	35 (28.22%)
Minimal residual disease	17 (13.71%)
Gross residual disease	72 (58.06%)

### Intra- and inter-observer reproducibility of feature extraction

The intra-observer ICC ranged from 0.802 to 0.923, and inter-observer ICC ranged from 0.761 to 0.902, which demonstrated that intra- and inter-observer reproducibility of radiomics feature extraction was agreeable.

### Radiomics signature screening

**Figure 2:**
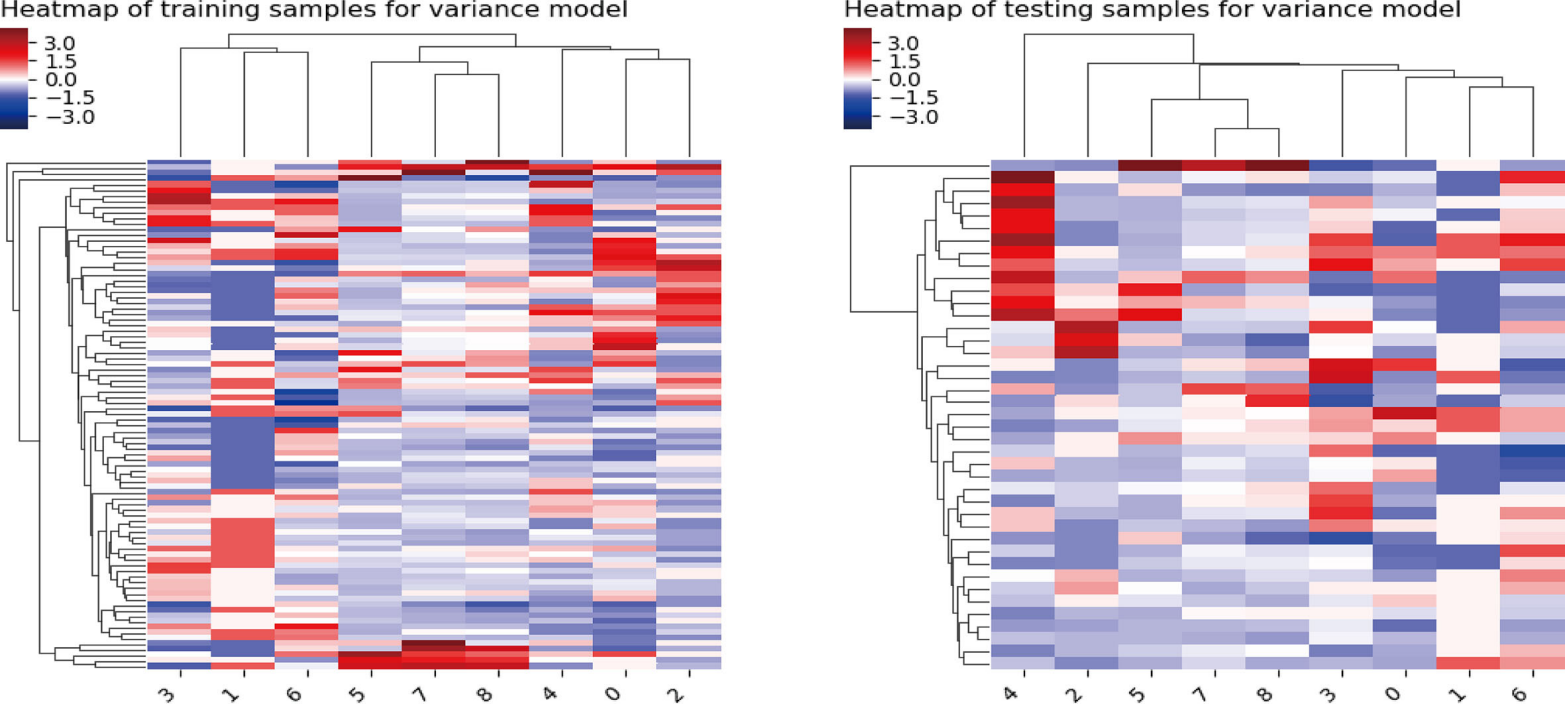
Heatmap of the model in the training and validation samples. For both the training samples and the validation samples, the numbers on the *x*-axis stand for different parameters; right to left represent Kurtosis, Gray-Level Variance, Gray-Level Non-Uniformity, Large Area Emphasis, Coarseness, Long-Run Low Gray-Level Emphasis, Busyness, Joint Entropy, and Complexity.

In the training cohort, a sum of 2,632 radiomics features were extracted from each VOI (1,316 for CT, 1,316 for PET), including (i) first-order feature, (ii) shape feature, (iii) gray-level co-occurrence matrix (GLCM) feature, (iv) gray-level size zone matrix (GLSZM) feature, (v) gray-level run length matrix (GLRLM) feature, (vi) neighborhood gray tone difference matrix (NGTDM) feature, and (vii) 14 gray-level dependence matrix (GLDM) features. We finally screened out 2,162 features with 470 features excluded due to relatively poor reproducibility (ICC range: 0.76–0.79). Then, the optimized subsets of nine radiomics features were selected based on the univariate logistic regression analysis, the multivariate logistic regression analysis, and the LASSO method. The heatmap of the model in the training and validation samples is displayed in **Figure 2**.

### Radiomics model building and evaluation

**Figure 3:**
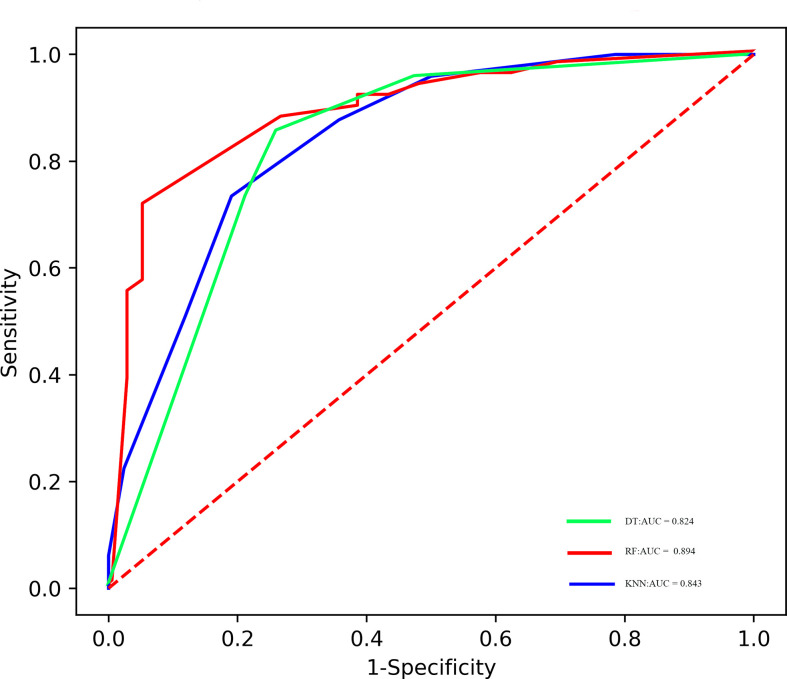
ROC of the different models in the training cohort.

**Figure 4:**
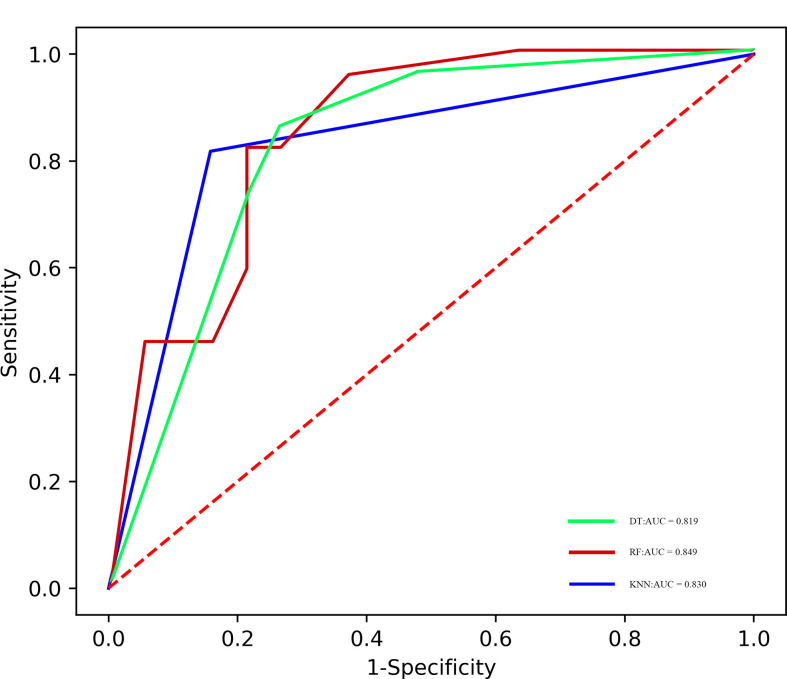
ROC of the different models in the validation cohort.

The predictive performance of radiomics models in the training and validation samples is shown in **Tables 3**, **4**, **5**, and corresponding ROCs of different models in the training and validation cohorts are demonstrated in **Figures 3** and **4**.

**Table 3:** Evaluation of the RF model in the training and validation samples.

Item	Training	Validation
Accuracy	0.824	0.805
Precision	0.946	0.818
AUC	0.894	0.849
Sensitivity	0.714	0.818
Specificity	0.952	0.789

**Table 4:** Evaluation of the DT model in the training and validation samples.

Item	Training	Validation
Accuracy	0.802	0.780
Precision	0.792	0.810
AUC	0.824	0.819
Sensitivity	0.857	0.773
Specificity	0.738	0.789

**Table 5:** Evaluation of the KNN in the training and validation samples.

Item	Training	Validation
Accuracy	0.769	0.829
Precision	0.818	0.857
AUC	0.843	0.830
Sensitivity	0.735	0.818
Specificity	0.810	0.842

### RF model

The areas under the curve (AUCs) of the RF model in the training set and validation set were 0.894 and 0.849, respectively. The accuracy, precision, sensitivity, and specificity were 0.824, 0.946, 0.714, and 0.952 in the training set, and 0.805, 0.818, 0.818, and 0.789 in the validation set, respectively.

### DT model

The AUCs of the RF model in the training set and validation set were 0.824 and 0.819, respectively. The accuracy, precision, sensitivity, and specificity were 0.802, 0.792, 0.857, and 0.738 in the training set, and 0.780, 0.810, 0.773, and 0.789 in the validation set, respectively.

### KNN model

The AUCs of the KNN model in the training set and validation set were 0.843 and 0.830, respectively. The accuracy, precision, sensitivity, and specificity were 0.769, 0.818, 0.735, and 0.810 in the training set, and 0.829, 0.857, 0.818, and 0.842 in the validation set, respectively.

## Discussion

In the present study, we demonstrated the clinical usefulness of radiomics features based on pretreatment ^18^F-FDG PET in predicting pathologic response to NAC treatment in BC patients. Three different machine learning classifiers, namely, KNN, RF, and DT, were developed in order to obtain the best diagnostic efficacy. In addition, glucose metabolic parameters and clinico-pathological parameters were incorporated into the radiomics model to optimize the predictive performance.

Tumor metabolic heterogeneity assessment based on ^18^F-FDG PET has been investigated previously (18). Regarding the non-invasive assessment of NAC pathological response using metabolic metrics derived from pretreatment ^18^F-FDG PET, in particular, the predictive potential of the primary tumor’s SUVmax was reported in various cancers. In a meta−analysis for FDG PET/CT, the predictive value of SUVmax was reported to have a pooled sensitivity of 0.847 and a pooled specificity of 0.661, which indicated that FDG PET/CT has reasonable sensitivity in assessing therapeutic efficacy to NAC in BC, which indicated that FDG PET/CT has reasonable sensitivity in assessing therapeutic efficacy to NAC in BC; however, the specificity is relatively low (19). However, champion et al. demonstrated that SUVmax on baseline PET scan, interim PET scan, and post-treatment PET scan did not statistically differ between the pCR and non-pCR groups. Meanwhile, our data provided concordant results to a previous study that SUVmax did not appear to be a predictor of pCR to NAC (20). On the basis of this fact, we guessed that the opposite findings observed may be caused by the intrinsic property of SUVmax as a PET parameter. SUVmax could be used to reflect only the most aggressive part instead of the entire tumor microenvironment. However, it is of vital significance to assess the tumor microenvironment due to the fact that the nonhomogeneous microenvironment perplexed the therapeutic response. Additionally, the intrinsic property of BC patients with relatively low FDG uptake at baseline or whose level of glucose metabolism cannot be altered by the NAC is not suitable for FDG PET-CT examination to evaluate the treatment effect. In our study, three standard PET/CT parameters, namely, SUVmax, SUVmean, and TLG, were analyzed; only baseline TLG was demonstrated to be a predictor of pCR to NAC treatment (*p* < 0.05), and was added to improve the radiomics model’s diagnostic power.

The ability of radiomics features derived from baseline ^18^ F-FDG PET to predict treatment response was recently reported in several works (21–24). In a previous study, Antunovic et al. developed a radiomics model by multiple logistic regression analysis to investigate the feasibility of using PET-CT radiomics analysis to assess the role of radiomics parameters in predicting pCR to NAC in patients with BC (25). However, firstly, it was carried out on a relatively small sample size (79 patients) and only PET-derived radiomics signatures were extracted. Secondly, the area under the curve value analysis for predicting therapeutic effect displayed limited discrimination performances (only ranging from 0.70 to 0.73), probably due to the small sample size, which was further reduced due to the missing data and the complete case approach used in the main analysis. Furthermore, advanced radiomics features were not extracted for all patients for technical reasons and there was a lack of validation set to assess the models’ diagnostic efficacy. In contrast, the AUCs of radiomics signatures ranged from 0.894 to 0.843 in the current study, which might provide a higher diagnostic performance. The current study used a relatively larger sample size, higher-order features, and advanced radiomics analysis methods, as well as high-dimensional radiomics signatures extracted up to 2,632. Its related engineering features were crucial for high-dimensional radiomics to avoid overfitting. Eventually, only optimal parameters were chosen to set up a diagnostic model. Overfitting is an inevitable issue that resulted from the high dimensionality of the radiomic signatures; thus, the population was randomly assigned in a 7:3 ratio to either the training cohort or the validation cohort to alleviate this problem. A previous study was designed to assess the clinical usefulness of textural signatures for predicting pCR to NAC. They found that the early changes in the textural signatures based on ^18^F-FDG PET images are predictive of pCR (26). The inconsistency between these results and our own specifically lies in the study cohort and in the approaches utilized for the analysis. Additionally, only SUV histogram (skewness), NGLCM (entropy), and NGTDM (coarseness) were available for textural analysis in the above study. Neither D %MTV nor D %TLG was an independent predictor of pCR in any group. It should be noted that the HER2-positive group is more likely to have a pCR to NAC than the HER2-negative group in our study, which agreed with previous works. In the HER2-positive group, all patients gained a greater benefit from trastuzumab. Baseline TLG demonstrated a potential predictive ability in our research, which is in line with the research results of Chen et al. They reported that pretreatment TLG can differentiate pCR from non-pCR to NAC in spite of the fact that the BC subtype was not mentioned. TLG represents the overall glucose metabolism level of tumor, which is related to the active level of tumor cell proliferation (27). Although all the data came from BC patients, there were still differences in the differentiation degree of tumor cells. The higher the level of glucose metabolism, the more active the proliferation of tumor cells, and the less the probability of pCR. However, some studies obtained discrepant findings. Lemarignier et al. confirmed that baseline TLG showed no predictive value in pCR assessment in BC patients (28). Discrepancies between studies may be due to the relatively small sample size and the limited number of events (i.e., pCR).

Ki-67 is a nuclear protein related to cell division and proliferation, which plays a key role in malignant tumor occurrence and development. Most NAC drugs can inhibit tumor cell proliferation and induce tumor cell apoptosis; thereby, tumor cell proliferation slows down, and the expression of Ki-67 decreases (29). Other investigators also have confirmed the clinical usefulness of Ki-67 as a predictive marker in the NAC response assessment, and they reported that a high pretreatment Ki-67 value instead of a low one was correlated with a higher pCR rate (30). Consistent with the previous study, our data also demonstrated the role of pretherapeutic Ki-67 as a predictive marker of pCR to NAC.

Currently, many different types of machine learning approaches can be applied to radiomics analysis; in this work, we constructed three multivariable classifiers, namely, KNN, DT, and RF, using pretreatment radiomics features of the primary tumor to predict pathological response to NAC, and we found that the RF model demonstrated the highest diagnostic performance (AUC of 0.894 *vs*. 0.843 *vs*. 0.824 in the training cohort; AUC of 0.849 *vs*. 0.830 *vs*. 0.819 in the validation cohort). The possible reasons are as follows: On the one hand, the RF machine learning algorithm is an outcome-driven machine learning approach and is composed of a set of decision trees, each of which is trained with randomly selected training data, and a random subset of radiomics signatures was applied to make decisions. Therefore, the data randomness guarantees low relevance and high diversity among the decision trees of the RF, which, in turn, ensures high stability in dealing with data disturbance and model generalizability (31). On the other hand, the RF algorithm also promotes the derivation of the prognostic factor. It is an inevitable truth that the number of algorithm calculations has increased exponentially due to the high-dimensional feature space. The RF machine learning algorithm is capable of selecting discriminative features from each cluster to build the radiomics model based on consensus clustering. Thus, the overall performance of the RF algorithm is better than other classifiers (32). Furthermore, no obvious difference in AUC values of all models between the training and the validation sets was observed; the possible reason might be that disadvantages such as overfitting and unbalanced data distribution are avoided in this study.

To summarize, our study still has some limitations. Firstly, although PET/CT-based radiomics analysis demonstrated a favorable performance in predicting the efficacy of NAC therapies, controversy still exists in relation to the application of PET/CT in clinical practice, mainly because of its high cost. However, we believed that potential savings are also associated with PET-CT scans as a result of avoiding additional imaging examinations or invasive procedures and by helping clinicians make the optimal treatment decisions. Secondly, although the final results achieved are ideal, the study population was still limited; a prospective study with a greater sample size should be conducted to further demonstrate our results. Thirdly, all the data were obtained from one single center; a multicenter trial with a much larger study cohort deserves further investigation in the near future. Lastly, tumor lesions were segmented using the manual method; an automated approach can be used to provide higher stability.

## Conclusion

In conclusion, we demonstrated that radiomics analysis based on pretreatment ^18^F FDG PET/CT scans can predict treatment response to NAC in BC. This approach shows great prospect for the early assessment of therapeutic effect non-invasively and accurately, which could potentially facilitate personalized precision medicine and avoid unnecessary treatment.

## Data availability statement

The raw data supporting the conclusions of this article will be made available by the authors, without undue reservation.

## Ethics statement

The studies involving human participants were reviewed and approved by Harbin Medical University Cancer Hospital. The ethics committee waived the requirement of written informed consent for participation.

## Author contributions

Conception and design: LY and JC. Collection and assembly of the data: XH and MP. Development of the methodology: YZ and TW. Data analysis and interpretation: PX and WC. Manuscript writing: All authors. Manuscript review: SC and SK. All authors contributed to the article and approved the submitted version.

## Funding

This paper is supported by the Haiyan Funding of Harbin Medical University Cancer Hospital (JJQN2019-23) (LY). The funder had no role in study design, data collection and analysis, decision to publish, or preparation of the manuscript.

## Conflict of interest

The authors declare that the research was conducted in the absence of any commercial or financial relationships that could be construed as a potential conflict of interest.

## Publisher’s note

All claims expressed in this article are solely those of the authors and do not necessarily represent those of their affiliated organizations, or those of the publisher, the editors and the reviewers. Any product that may be evaluated in this article, or claim that may be made by its manufacturer, is not guaranteed or endorsed by the publisher.

## Glossary

**Table:**

^18^F-FDG	^18^F-fluorodeoxyglucose
PET/CT	positron emission tomography
NAC	neoadjuvant chemotherapy
LABC	locally advanced breast cancer
VOI	volume of interest
Pcr	pathological complete response
AUC	area under the curve
RF	random forest
DT	decision tree
KNN	k-nearest neighbors
BC	breast cancer
DFS	disease-free survival
Her-2	human epidermal growth factor receptor 2
OS	overall survival
SUV max	maximum standardized uptake value
ICCs	intra- and inter-class correlation coefficients
LASSO	least absolute shrinkage and selection operator
ROC	receiver operating characteristic
TLG	total lesion glycolysis
GLCM	gray-level co-occurrence matrix
GLSZM	gray-level size zone matrix
GLRLM	gray-level run length matrix
NGTDM	neighborhood gray tone difference matrix
GLDM	gray-level dependence matrix
GLNU	gray-level non-uniformity
ER	estrogen receptor
PR	progesterone receptor
CEA	carcinoembryonic antigen
CA153	carbohydrate antigen 153
