# Supplementary material for: PET/CT-based radiomics analysis may help to predict neoadjuvant chemotherapy outcomes in breast cancer
Source: Front Oncol. 2022 Nov 7;12:849626. doi: 10.3389/fonc.2022.849626 (PMC9676961; doi:10.3389/fonc.2022.849626)
Supplement: Supplementary file 1 [file DataSheet_1.pdf]

Supplemental Data

Supplementary Table 1. Results of univariate and multivariate logistic regression analysis.

Variables	Univariate analysis OR (95% CI)	*P*	Multivariate analysis OR (95% CI)	*P*
Sex	1.008 (0.984-1.021)	0.813	1.023 (0.974-1.098)	0.724
Age	1.105 (0.978-1.021)	0.716	1.008 (0.958-1.075)	0.622
CEA	1.054 (0.992-1.035)	0.602	1.002 (0.970-1.041)	0.304
CA153	0.986 (0.963-1.019)	0.845	0.904 (0.832-0.977)	0.362
ER	0.981 (0.974-1.001)	0.467	0.987 (0.944-1.010)	0.456
Histology	1.025 (0.720-1.089)	0.398	0.985 (0.940-1.016)	0.440
Ki 67	0.954 (0.916-0.969)	$P < 0.05$	0.962 (0.921-0.987)	$P < 0.05$
pN stage	1.125 (1.058-1.366)	0.279	1.6142 (1.048-3.410)	0.169
PR	0.980 (0.942-1.029)	0.498	1.009 (0.976-1.058)	0.599
SUVmax	1.019 (0.970-1.072)	$P < 0.05$	0.912 (0.838-1.062)	0.164
SUVmean	1.022 (0.974-1.072)	0.268	0.993 (0.964-1.022)	0.788
TLG	1.021 (1.013-1.027)	$P < 0.05$	0.996 (0.938-1.044)	$P < 0.05$
pT stage	0.466 (0.424-1.4581)	0.202	0.310 (0.092-0.564)	0.137
Tumor grade	0.947 (0.923-0.955)	$P < 0.05$	1.247 (1.164-1.471)	$P < 0.05$

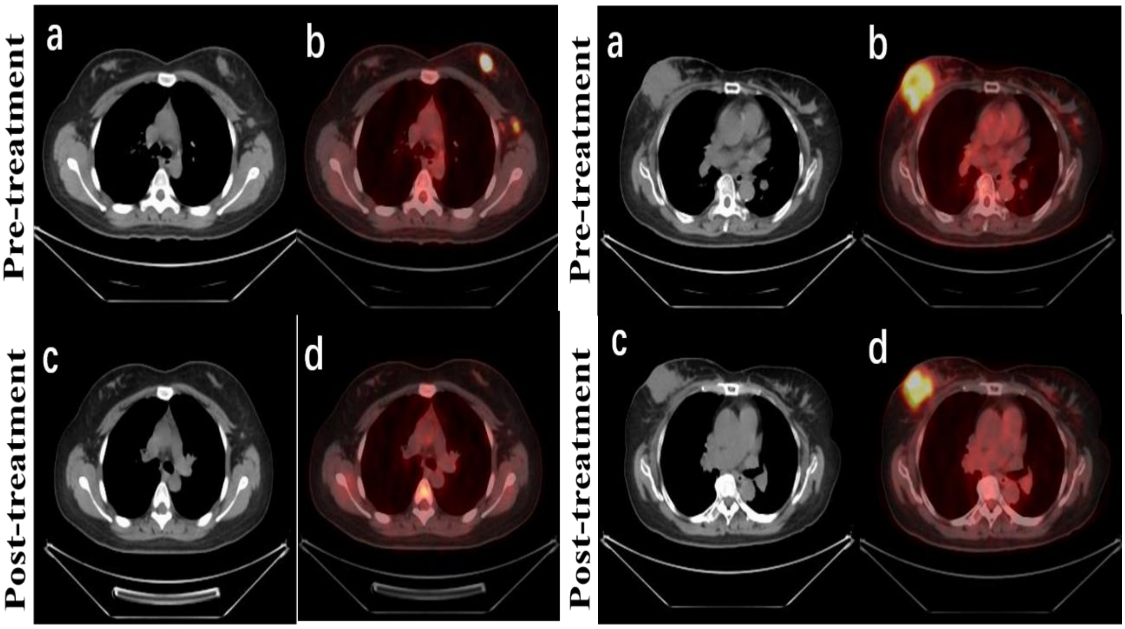

Supplementary Figure 1. A: Representative PET/CT images of a patient with pCR after NAC. (**a**) pre-treatment axial CT image and (**b**) fused axial PET/CT image, (**c**) post-treatment axial CT image and (**d**) fused axial PET/CT image in a 61-year-old patient with HER2+ breast cancer, with no evidence of residual disease at postsurgical histological analysis after neoadjuvant treatment. **B**: Representative PET/CT images of a patient with pathological residual disease after NAC. (**a**) pre-treatment axial CT image and (**b**) fused axial PET/CT image, (**c**) post-treatment axial CT image and (**d**) fused axial

PET/CT image in a 42-year-old patient with HER2+ breast cancer, with persistence of disease at postsurgical histological analysis after neoadjuvant treatment.

Supplementary Material 3 Radiomics Features

This section contains the definitions of the various features that can be extracted using PyRadiomics. They are subdivided into the following classes:

• First Order Statistics (19 features)

• Shape-based (16 features)

• Gray Level Cooccurence Matrix (24 features)

• Gray Level Run Length Matrix (16 features)

• Gray Level Size Zone Matrix (16 features)

• Neigbouring Gray Tone Difference Matrix (5 features)

• Gray Level Dependence Matrix (14 features)

Specific radiomic features are listed as follows:

- **First Order Statistics (19 features)**

1. Energy
2. Total Energy
3. Entropy
4. Minimum
5. 10th percentile
6. 90th percentile
7. Maximum
8. Mean
9. Median
10. Interquartile Range
11. Range

12. Mean Absolute Deviation (MAD)

13. Robust Mean Absolute Deviation (rMAD)

14. Root Mean Squared (RMS)

15. AbsoluteDeviation

16. Skewness

17. Kurtosis

18. Variance

19. Uniformity

- **Shape-based (14 features)**

1. Flatness
2. Least Axis Length
3. Major Axis Length
4. Maximum 2D DiameterColumn
5. Maximum 2D DiameterRow
6. Maximum 2D DiameterSlice
7. Maximum 3D Diameter
8. MeshVolume
9. Minor Axis Length
10. Sphericity
11. SurfaceArea
12. Surface Volume Ratio
13. Voxel Volume
14. Elongation

- **Gray Level Cooccurence Matrix (24 features)**

1. Autocorrelation
2. joint Average
3. Cluster Prominence
4. Cluster Shade

5. Cluster Tendency
6. Contrast
7. Correlation
8. Difference Entropy
9. Difference Variance
10. Difference Average
11. Joint Energy
12. Joint Entropy
13. Informational Measure of Correlation (IMC) 1
14. Informational Measure of Correlation (IMC) 2
15. Inverse Difference Moment (IDM
16. Maximal Correlation Coefficient (MCC
17. Inverse Difference Moment Normalized (IDMN)
18. Inverse Difference (ID)
19. Inverse Difference Normalized (IDN)
20. Inverse Variance
21. Maximum Probability
22. Sum Average
23. Sum Entropy
24. Sum of Squares

- **Gray Level Run Length Matrix (16 features)**

1. Short Run Emphasis (SRE)
2. Long Run Emphasis (LRE)
3. Gray Level Non-Uniformity (GLN)
4. Gray Level Non-Uniformity Normalized (GLNN)
5. Run Length Non-Uniformity (RLN)
6. Run Length Non-Uniformity Normalized (RLNN)
7. Run Percentage (RP)

8. Gray Level Variance (GLV)
9. Run Variance (RV)
10. Run Entropy (RE)
11. Low Gray Level Run Emphasis (LGLRE)
12. High Gray Level Run Emphasis (HGLRE)
13. Short Run Low Gray Level Emphasis (SRLGLE)
14. Short Run High Gray Level Emphasis (SRHGLE)
15. Long Run Low Gray Level Emphasis (LRLGLE)
16. Long Run High Gray Level Emphasis (LRHGLE)

- **Gray Level Size Zone Matrix (16 features)**

1. Small Area Emphasis (SAE)
2. Large Area Emphasis (LAE)
3. Gray Level Non-Uniformity (GLN)
4. Gray Level Non-Uniformity Normalized (GLNN)
5. Size-Zone Non-Uniformity (SZN)
6. Size-Zone Non-Uniformity Normalized (SZNN)
7. Zone Percentage (ZP
8. Gray Level Variance (GLV)
9. Zone Variance (ZV)
10. Zone Entropy (ZE)
11. Low Gray Level Zone Emphasis (LGLZE)
12. High Gray Level Zone Emphasis (HGLZE)
13. Small Area Low Gray Level Emphasis (SALGLE)
14. Small Area High Gray Level Emphasis (SAHGLE)
15. Large Area Low Gray Level Emphasis (LALGLE)
16. Large Area High Gray Level Emphasis (LAHGLE)

- **Neigbouring Gray Tone Difference Matrix (5 features)**

1. Busyness

2. Coarseness
3. Complexity
4. Contrast
5. Strength

- **Gray Level Dependence Matrix (14 features)**

1. Small Dependence Emphasis (SDE)
2. Large Dependence Emphasis (LDE)
3. Gray Level Non-Uniformity (GLN)
4. Dependence Non-Uniformity (DN)
5. Dependence Non-Uniformity Normalized (DNN)
6. Gray Level Variance (GLV
7. Dependence Variance (DV)
8. Dependence Entropy (DE)
9. Low Gray Level Emphasis (LGLE)
10. High Gray Level Emphasis (HGLE)
11. Small Dependence Low Gray Level Emphasis (SDLGLE)
12. Small Dependence High Gray Level Emphasis (SDHGLE)
13. Large Dependence Low Gray Level Emphasis (LDLGLE)
14. Large Dependence High Gray Level Emphasis (LDHGLE)

Specific parameters used to build the model

The machine learning method: KNN

the parameters selection method and selected parameters:

{'n_neighbors': 22, 'Method': 'manual'}

the separate scores and total mean scores of model in each validation fold:

{'scores': array ([0.61904762, 0.71428571, 0.80952381, 0.9, 0.78947368]), 'mean_score': 0.7664661654135339}

The machine learning method: RF

the parameters selection method and selected parameters:

{'n_estimators': 11, 'max_depth': 1, 'Method': 'manual', 'min_samples_split': 5}

the separate scores and total mean scores of model in each validation fold:

{'scores': array ([0.61904762, 0.66666667, 0.85714286, 0.75, 0.42105263]), 'mean_score': 0.662781954887218}

The machine learning method: DT

the parameters selection method and selected parameters:

{'kernel': 'linear', 'C': 0.1, 'Method': 'manual'}

the separate scores and total mean scores of model in each validation fold:

{'scores': array ([0.53333333, 0.52272727, 0.51162791, 0.62790698, 0.55813953]), 'mean_score': 0.5507470049330514}

The coefficient of selected features

**Variables**	**Coef.**
Kurtosis	0.4406
GrayLevelVariance	2.0487
GrayLevelNonUniformity	-1.3269
LargeAreaEmphasis	1.3317
Coarseness	-0.9155
LongRunLowGrayLevelEmphasis	-0.4075
Busyness	0.8441
JointEntropy	1.0612
Complexity	1.0225
